# The Effects of the A Matter of Balance Program on Falls and Physical Risk of Falls, Tampa, Florida, 2013

**DOI:** 10.5888/pcd12.150096

**Published:** 2015-09-24

**Authors:** Tuo-Yu Chen, Jerri D. Edwards, Megan C. Janke

**Affiliations:** Author Affiliations: Jerri D. Edwards, School of Aging Studies, University of South Florida, Tampa, Florida; Megan C. Janke, Department of Recreation and Leisure Studies, East Carolina University, Greenville, North Carolina.

## Abstract

**Introduction:**

This study investigated the effects of the A Matter of Balance (MOB) program on falls and physical risk factors of falling among community-dwelling older adults living in Tampa, Florida, in 2013.

**Methods:**

A total of 110 adults (52 MOB, 58 comparison) were enrolled in this prospective cohort study. Data on falls, physical risk of falling, and other known risk factors of falling were collected at baseline and at the end of the program. Multivariate analysis of covariance with repeated measures and logistic regressions were used to investigate the effects of this program.

**Results:**

Participants in the MOB group were less likely to have had a fall and had significant improvements in their physical risk of falling compared with adults in the comparison group. No significant effects of the MOB program on recurrent falls or the number of falls reported were found.

**Conclusion:**

This study contributes to our understanding of the MOB program and its effectiveness in reducing falls and the physical risk of falling among older adults. The findings support extended use of this program to reduce falls and physical risk of falling among older adults.

## Introduction

The A Matter of Balance (MOB) program is a multicomponent cognitive-behavioral intervention (1,2). The program was designed to reduce fear of falling by enhancing falls self-efficacy and perceived control over falling and to promote continued safe engagement in activity (1,2). Its curriculum incorporates standardized behavioral education (eg, risk behaviors of falling, environmental hazards) and exercise (ie, balance and strength training) components. Each session covers an educational topic, and exercise is introduced to participants during the third session and practiced at the beginning of each subsequent session (1).

Although the MOB program has been widely provided to community-dwelling older adults, the reported effects of the MOB program on falls are inconsistent. Furthermore, the effects on physical risk of falling are not known. Four studies have reported the effects of the MOB program on falls (1–4) as indicated by number of fallers (ie, number who fell at least once), recurrent fallers (ie, number who fell 2 or more times), and total falls (ie, counts of falls). Two randomized controlled trials (1,2) indicate that the MOB program reduces the number of recurrent fallers but not the number of fallers or total falls. Two single-group studies (3,4) indicate that the MOB program significantly reduces the number of falls, but the number of fallers or recurrent fallers was not examined. Similarly, the effect of the MOB program on physical risk factors of falling is not well documented. Only 1 study using a single-group design (5) addressed this effect.

The objective of this study was to compare falls status and physical risk factors of falling between MOB and comparison groups to further our understanding of the effects of the program on falls and physical risk factors of falling.

## Methods

This study used a quasi-experimental design (pretests and posttests with MOB and comparison groups). The institutional review board at the University of South Florida approved the study.

**Figure 1:**
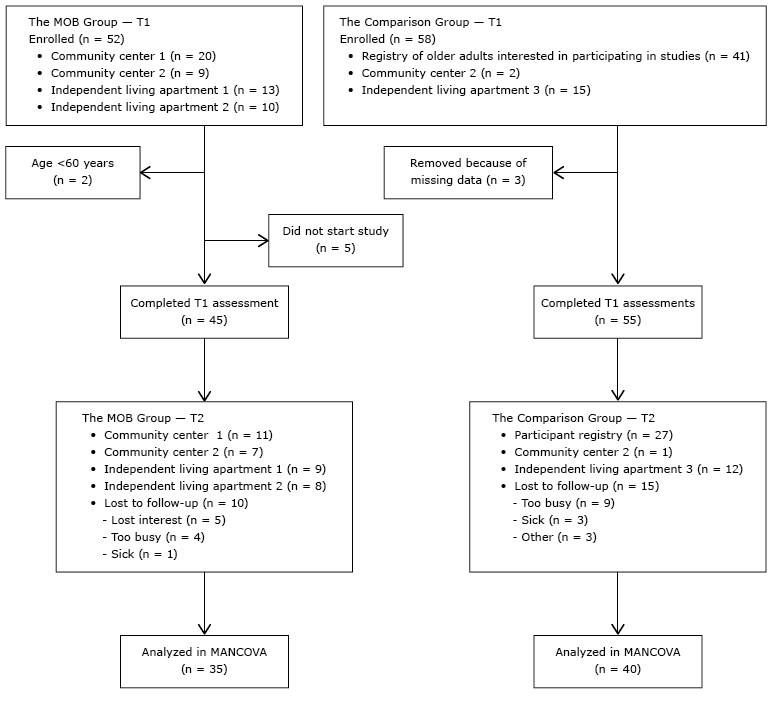
Process for including participants in the A Matter of Balance (MOB) group and the comparison group in the analysis, Tampa, Florida, 2013. Abbreviations: T1, Time 1; T2, Time 2; MANCOVA, multivariate analysis of covariance.

Participants in Tampa, Florida, were required to be community-dwelling adults and at least 60 years of age, understand English, and not use a wheelchair. Fifty-two participants were recruited for the MOB group through flyers in 2 community centers and 2 independent living apartments where the program was offered. None of these individuals had previously participated in the MOB program. For the comparison group, 58 individuals were recruited through flyers posted in the same locations and a registry for older adults who were interested in participating in studies (Figure 1).

The standardized MOB program was provided by the West Central Florida Area Agency on Aging. The program took place once per week for 8 weeks (2). Individuals in the comparison group received no intervention but completed assessments at the same time points as the MOB group.

### Measures

The outcome variables for this study were falls and physical risk of falling. Fall-related risk factors were also assessed (6–8).

A fall was defined as “an unexpected event in which participants come to rest on the ground, floor, or lower level” (9). Participants were asked, “Have you experienced any falls in the past two months?” Individuals who answered yes were asked, “How many times did you fall in the past two months?” Three variables were created: 1) the number of falls, 2) fallers (fallers coded as 1, nonfallers coded as 0), and 3) recurrent fallers (recurrent fallers coded as 1, nonrecurrent fallers coded as 0).

Participants were asked to perform 3 physical measures commonly used to predict falls. The Performance-Oriented Mobility Assessment (POMA) (10) consisted of balance and gait components. A combined score (range, 0–28) was calculated for each individual; lower scores indicated a higher likelihood of falling. The Timed Up and Go (TUG) test (6) measured the time (in seconds) to walk a 3-meter course at a normal pace, starting from and ending in the same chair; longer times indicated a higher likelihood of falling. The Functional Reach (FR) test (11) assessed the distance (in inches) that individuals could reach forward without moving their feet; shorter distances indicated greater likelihood of falling.

Information about age, sex, race, and education was obtained at Time 1 (T1). Age and education were recorded in years. Female was coded as 1 and male as 0. Race was coded as follows: white, 1; Hispanic, 2; black, 3; and Asian, 4.

Participants were asked “Are you often troubled with pain?” (1 = trouble with pain most of the time, 0 = no trouble with pain). Participants who reported some pain, but noted that it did not affect them, were coded as 0. Participants were asked if they had any of these conditions diagnosed: high blood pressure, diabetes, cancer, lung disease, stroke, arthritis, depression, heart disease, osteoporosis, or asthma (1 = yes, 0 = no, for each condition). The composite score ranged from 0 to 10; higher scores indicated more chronic conditions. The Katz Activities of Daily Living scale (12) and the Lawton Instrumental Activities of Daily Living scale (13) were used to assess functional limitations (1 = dependent, 0 = independent). The composite score ranged from 0 to 14 (14); higher scores indicated more functional limitations. The Montreal Cognitive Assessment (MoCA) was used to assess overall cognitive status (15). The total score ranged from 0 to 30; higher scores indicated better cognitive status.

Research indicates that fear of falling and falls efficacy should be separately measured (16). Therefore, the geriatric fear of falling measure (GFFM) (17) was used to measure fear of falling and the Modified Falls Efficacy Scale (MFES) (18) was used to assess falls efficacy. The GFFM includes 15 statements (eg, “I go out less during rainy days.”). Participants rated their level of agreement for each statement from 1 (never) to 5 (always). The total score ranged from 15 to 75; higher scores indicated greater fear of falling (Cronbach’s α = 0.85–0.91; test–retest reliability *r* = 0.78). The concurrent validity with the MFES was *r* = −0.73. The MFES includes 14 activities. Participants were asked to rate their level of confidence (0 = not confident, 10 = completely confident) in completing activities (eg, crossing road) without falling. Participants were asked to rate activities hypothetically if they did not perform them. The average score ranged from 0 to 10; higher scores indicated more confidence in performing activities without falling.

Number of physical activities was recorded at T1 to ensure that there were no differences between the groups. During the interviews, participants were asked, “Do you currently participate in any exercise group or program?” Those who answered yes were then asked, “How many exercise groups or programs are you participating in each week?” The number of physical activities reported was used in analyses.

### Procedure

Informed consent was obtained. For the MOB group, the T1 assessment took place 1 week before the start of the program, and the Time 2 (T2) assessment was completed within 2 weeks of the final MOB session. The duration between the 2 assessments was similarly scheduled for the comparison group. Each interview lasted approximately 40 minutes.

### Analyses

First, multivariate analysis of variance (MANOVA) was used to examine attrition. Next, MANOVA (for continuously scaled outcomes) and χ^2^ statistics (for categorically scaled outcomes) were performed to investigate significant differences in characteristics between the 2 groups at T1. Variables significantly different between the 2 groups at T1 were used as covariates in subsequent analyses to examine the effects of the MOB program. Third, a 2 × 2 (group × time) repeated-measures multivariate analysis of covariance (MANCOVA) (19) was used to compare the MOB and comparison groups from T1 to T2 across all continuously scaled dependent variables (the number of falls, the POMA, the TUG test, and the FR test) adjusting for covariates at T1. A significant multivariate group × time interaction was followed by univariate repeated-measures analyses of covariance (ANCOVAs) to examine which dependent variable(s) reflected significant differences between the groups from T1 to T2. Significant univariate group × time interactions were followed by Fisher least significant difference (LSD) tests to make comparisons between and within groups. Last, logistic regressions were performed to examine the effects of the MOB program on categorical variables of falls (ie, fallers and recurrent fallers) at T2 accounting for covariates at T1. A *P* level of less than .05 was considered as significant in all analyses.

## Results

Results from the MANOVA examining attrition showed no significant differences at T1 between participants who did and did not complete the study in the MOB group (Wilks’ Λ = 0.70; *F*_12,32_ = 1.17; *P* = .34; η^2^ = 0.30) or in the comparison group (Wilks’ Λ = 0.83; *F*_12,42_ = 0.71; *P* = .73; η^2^ = 0.17). 

MANOVA identified some significant differences between the 2 groups at T1 (Wilks’ Λ = 0.76; *F*_8,91_ = 3.61; *P* = .001; η^2^ = 0.24). Follow-up univariate ANOVAs revealed significant differences between the 2 groups at T1 in age, number of chronic conditions, number of functional limitations, MoCA scores, the GFFM, and the MFES (Table 1). The χ^2^ statistics showed a significant difference in race between the 2 groups (*P* = .001). These variables were included as covariates in the subsequent analyses.

**Table 1:** Characteristics of the Matter of Balance (MOB) and Comparison Groups at Time 1^a^, Tampa, Florida, 2013

Variable	MOB (n = 45)	Comparison (n = 55)	*F*_1,98_ or χ^2^ (N = 100)	*P*	η^2^
**Age, mean (SD), y**	78.9 (9.3)	74.8 (8.2)	8.53	.004	0.08
**Female sex, %**	76	71	0.27	.60	—
**Race, %**					
White	64	96	17.20^b^	.001	—
Hispanic	31	4			
Black	2	0			
Asian	2	0			
**Education, mean (SD), y**	14 (3.3)	15 (2.2)	2.95	.08	0.03
**Pain, %**	73	64	1.07	.30	—
**Chronic conditions (0**–**10), mean (SD), n**	3.3 (1.6)	2.4 (1.6)	7.29	.008	0.16
**Functional limitations (0**–**14),^c^ mean (SD), n**	1.9 (2.8)	0.8 (2.2)	5.06	.02	0.05
**Montreal Cognitive Assessment (0**–**30), mean (SD), score**	22.1 (5.4)	26.0 (3.7)	17.98	<.001	0.16
**Geriatric fear of falling measure (15–75), mean (SD), score**	39.5 (12.0)	31.2 (10.8)	13.33	<.001	0.12
**Modified Falls Efficacy Scale (0**–**10), mean (SD)**	7.2 (2.3)	8.5 (1.7)	11.55	.001	0.11
**Number of exercise groups or programs/week, mean (SD)**	1.0 (1.3)	1.2 (1.4)	0.43	.51	<0.01

Abbreviation: —, not applicable; SD, standard deviation.

a Multivariate analysis of variance was used to examine the differences in continuous variables between the MOB group and the comparison group. χ^2^ statistics were used for categorical variables.

b The degrees of freedom is 3.

c The Katz Activities of Daily Living scale (12) and the Lawton Instrumental Activities of Daily Living scale (13) were used to assess functional limitations (1 = dependent, 0 = independent). The composite score ranged from 0 to 14 (14); higher scores indicated more functional limitations.

Table 2 shows the unadjusted scores of the outcome variables. The multivariate analysis showed a significant main effect of group (Wilks’ Λ = 0.79; *F*_4,63_ = 4.13; *P* = .005; η^2^ = 0.21), no significant effect of time (Wilks’ Λ = 0.94; *F*_4,6_ = 1.06; *P* = .38; η^2^ = 0.06), and a significant group × time interaction (Wilks’ Λ = 0.53; *F*_4,63_ = 13.79; *P* < .001; η^2^ = 0.47).

**Table 2:** Outcome Variables Between the Matter of Balance (MOB) Group and Comparison Group (Unadjusted)^a^, Tampa, Florida, 2013

Variable	MOB	Comparison	*P*
Fallers (T1),^b^ n (%)	15 (33)	16 (29%)	.65
Fallers (T2),^b^ n (%)	4 (11)	12 (30%)	.05
Recurrent fallers (T1),^c^ n (%)	4 (8.9)	5 (9.1%)	.97
Recurrent fallers (T2),^c^ n (%)	1 (2.9)	4 (10%)	.22
Number of falls (T1)^b^	19	23	.97
Number of falls (T2)^b^	5	19	.04
Performance-Oriented Mobility Assessment score (0–28; T1),^d^ mean (SD)	22.8 (4.1)	24.75 (3.9)	.02
Performance-Oriented Mobility Assessment score (0–28; T2),^d^ mean (SD)	24.05 (4.0)	23.6 (4.8)	.67
Timed Up and Go test (seconds) (T1),^b^ mean (SD)	14.3 (3.6)	12.59 (4.0)	.03
Timed Up and Go test (seconds) (T2),^b^ mean (SD)	12.87 (3.5)	14.0 (5.7)	.29
Functional Reach test (inches) (T1),^d^ mean (SD)	9.3 (2.4)	11.5 (2.3)	<.001
Functional Reach test (inches) (T2),^d^ mean (SD)	10.5 (2.3)	10.6 (2.2)	.76

Abbreviations: T1, Time 1; T2, Time 2; SD, standard deviation

a At T1, there were 45 individuals in the MOB group and 55 in the comparison group. At T2, there were 35 individuals in the MOB group and 40 in the comparison group. Independent *t* tests and χ^2^ statistics were used for continuous and categorical variables, respectively.

b Lower is better.

c Number of people who fell 2 or more times.

d Higher assessment scores are better.

 For the number of falls, the univariate repeated-measures ANCOVA showed a significant main effect of group (*F*_1,66_ = 9.45; *P* = .003; η^2^ = 0.12), but no effect of time and no group × time interaction (Table 3). The nonsignificant group × time interaction indicated that the number of falls did not change significantly from T1 to T2 between the MOB and comparison groups.

**Table 3:** Univariate Repeated-Measures ANCOVAs for the Number of Falls, the Performance-Oriented Mobility Assessment (POMA), the Timed Up and Go (TUG) Test, and the Functional Reach (FR) Test, Tampa, Florida, 2013

Variables	Number of Falls^a^			POMA^b^			TUG Test^a^			FR Test^b^		
	*F* c	*P*	η^2^	*F* c	*P*	η^2^	*F* c	*P*	η^2^	*F* c	*P*	η^2^
**Group**	9.45	.003	0.12	2.53	.11	0.02	7.31	.009	0.08	0.05	.28	<0.01
**Time**	1.97	.16	0.02	1.34	.25	0.01	0.60	.44	0.01	1.01	.31	0.01
**Group × Time**	1.80	.18	0.02	21.38	<.001	0.22	21.14	<.001	0.23	24.07	<.001	0.25
**Covariates**												
Age, y	0.63	.43	—	16.74	<.001	—	8.22	.006	—	11.20	.001	—
Race, %	0.35	.55	—	1.69	.19	—	0.36	.55	—	0.59	.45	—
CC (score range, 0–10)	0.71	.40	—	4.34	.04	—	2.90	.09	—	0.56	.45	—
FL^d^ (score range, 0–14)	0.15	.69	—	3.46	.06	—	5.80	.01	—	1.05	.30	—
MoCA (score range, 0–30)	0.35	.55	—	5.68	.02	—	0.92	.34	—	1.30	.25	—
GFFM (score range, 15–75)	0.20	.65	—	2.28	.13	—	5.53	.02	—	2.27	.13	—
MFES (score range, 0–10)	3.21	.07	—	8.67	.004	—	2.29	.13	—	2.22	.14	—

Abbreviations: — , not applicable; ANCOVA, analysis of covariance; CC, number of chronic conditions; FL, functional limitations; MoCA, Montreal Cognitive Assessment; GFFM, geriatric fear of falling measure; MFES, Modified Falls Efficacy Scale.

a Lower is better.

b Higher is better.

c The degrees of freedom is 1,66.

d The Katz Activities of Daily Living scale (12) and the Lawton Instrumental Activities of Daily Living scale (13) were used to assess functional limitations (1 = dependent, 0 = independent). The composite score ranged from 0 to 14 (14); higher scores indicated more functional limitations.

**Figure 2:**
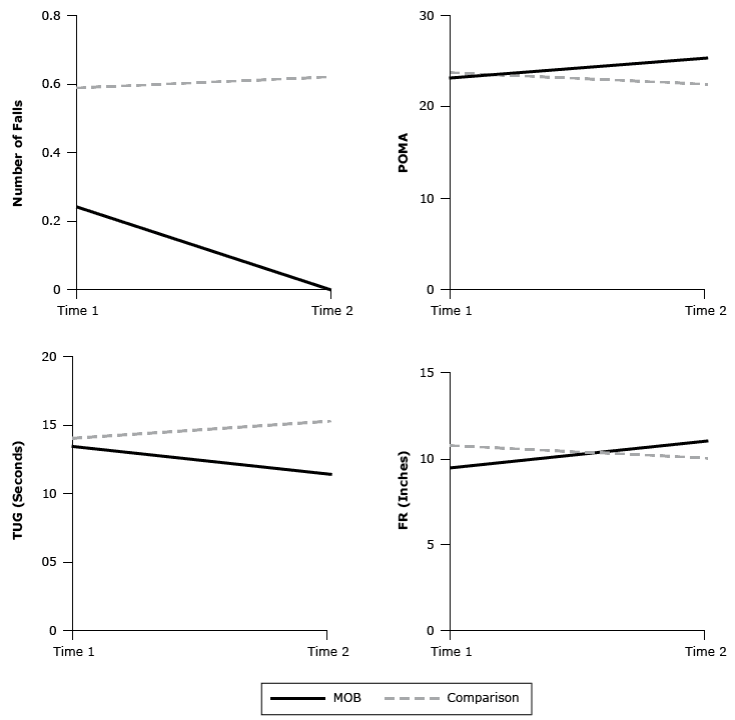
Comparisons of group × time interactions for the number of falls, the Performance-Oriented Mobility Assessment (POMA), the Timed Up and Go (TUG) test, and the Functional Reach (FR) test for participants in the A Matter of Balance (MOB) program and a comparison group, Tampa, Florida, 2013. All outcome variables were adjusted for covariates (age, race, chronic conditions, functional limitations, Montreal Cognitive Assessment scores, geriatric fear of falling measure scores, and Modified Falls Efficacy Scale scores) at Time 1. For the number of falls and the TUG test, lower numbers are better. For the POMA and the FR test, higher numbers are better. For POMA, TUG test, and FR test, differences between the MOB and comparison group were significant, *P* < .001. **Table:** 

For the POMA, the univariate repeated-measures ANCOVA showed no main effect of group or time, but the group × time interaction was significant (*F*_1,66_ = 21.38; *P* < .001; η^2^ = 0.22) (Table 3). Fisher LSD tests revealed no significant differences in the POMA between the MOB and comparison groups at T1 (*P* = .62). However, the MOB group had significantly better performance than the comparison group at T2 (*P* = .002). In addition, from T1 to T2, the MOB group demonstrated a significant improvement on the POMA (*P* < .001), but the comparison group had significant declines (*P* = .01) (Figure 2).

Variable	Time 1, Mean	Time 2, Mean
**Number of falls**		
MOB	0.2	0
Comparison	0.6	0.6
**Performance-Oriented Mobility Assessment**		
MOB	23.3	25.3
Comparison	23.6	22.5
**Timed Up and Go test (seconds)**		
MOB	13.5	11.4
Comparison	14.1	15.3
**Functional Reach test (inches)**		
MOB	9.6	11.1
Comparison	10.8	10.1

For the TUG test, the univariate repeated-measures ANCOVA revealed a significant main effect of group (*F*_1,66_ = 7.31; *P* = .009; η^2^ = 0.08), no effect of time, and a significant group × time interaction (*F*_1,66_ = 21.14; *P* < .001; η^2^ = 0.23) (Table 3). Fisher LSD tests showed no significant differences in the TUG test between the 2 groups at T1 (*P* = .48). At T2, the MOB group performed significantly better on the test than the comparison group (*P* < .001). Furthermore, from T1 to T2, the MOB group improved significantly on the TUG test (*P* < .001) while the comparison group showed a decline (*P* = .007) (Figure 2).

For the FR test, the univariate repeated-measures ANCOVA indicated no main effect for group or time but a significant group × time interaction (*F*_1,66_ = 24.07; *P* < .001; η^2^ = 0.25) (Table 3). Fisher LSD tests revealed that the comparison group had significantly better performance on the FR test than the MOB group at T1 (*P* = .01). However, the MOB group performed better on the FR test than those in the comparison group at T2 (*P* = .04). Moreover, from T1 to T2, the MOB group improved significantly on the FR test (*P* < .001), but the comparison group experienced a decline (*P* = .01) (Figure 2).

Two logistic regressions were used to examine whether participants in the MOB program were less likely to be fallers and recurrent fallers at T2 accounting for covariates at T1 (Table 4). Significant factors associated with fallers at T2 included being in the MOB group (OR, 0.06; *P* = .01; 95% CI, 0.01–0.60) and having functional limitation (OR, 1.82; *P* = .03; 95% CI, 1.05–3.17). Participants in the MOB group were 84% less likely to report any fall at T2 than those in the comparison group. For recurrent fallers, participation in the MOB group was not a significant factor in reducing recurrent falls at T2 (OR, 0.06; *P* = .14; 95% CI, 0.002–2.52). No other significant factors were related to recurrent fallers at T2.

**Table 4:** Logistic Regressions Examining the Effects of the A Matter of Balance (MOB) Program on Falls and Recurrent Falls at Time 2, Tampa, Florida, 2013

Variables	Fallers	Recurrent Fallers
	OR (95% CI)	OR (95% CI)
MOB group	0.06^a^ (0.01–0.60)	0.06 (0.002–2.52)
Age, y	1.09 (0.98–1.20)	1.01 (0.85–1.21)
Race	1.26 (0.13–11.78)	0^b^
Number of chronic conditions (0–10)	0.79 (0.46–1.35)	0.90 (0.29–2.87)
Functional limitations^c^ (0–14)	1.82^a^ (1.05–3.17)	1.49 (0.79–2.82)
Montreal Cognitive Assessment (0–30)	1.16 (0.64–1.43)	1.00 (0.76–1.31)
Geriatric fear of falling measure (15–75)	1.01 (0.92–1.12)	0.97 (0.77–1.22)
Modified Falls Efficacy Scale (0–10)	0.96 (0.55–1.66)	0.53 (0.16–1.77)

Abbreviations: OR, odds ratio; CI, confidence interval.

a *P* < .05.

b All recurrent fallers at Time 2 were white.

c The Katz Activities of Daily Living scale (12) and the Lawton Instrumental Activities of Daily Living scale (13) were used to assess functional limitations (1 = dependent, 0 = independent). The composite score ranged from 0 to 14 (14); higher scores indicated more functional limitations.

## Discussion

Our study examined the effects of the MOB program on falls status and physical risks of falling. After accounting for covariates at T1, the analyses revealed that participants in the MOB group were less likely to be fallers at T2 than those in the comparison group. However, the MOB program appeared to have no significant impact on recurrent falls or the number of falls reported. The MOB group performed significantly better than the comparison group on the POMA, the TUG test, and the FR test across time, after adjusting for covariates. Given that the adults in the MOB group were older, had more chronic conditions, more functional limitations, worse global cognitive function, greater fear of falling, and lower falls efficacy than those in the comparison group at T1, these individuals were at higher risk for falls over time than the comparison group. Nevertheless, the older adults who participated in the MOB group had less physical risk of falls over time relative to the comparison group. Thus, the potential of the MOB program to reduce falls and physical risk factors of falling in the aging population is notable.

Unlike previous studies (2–4), this study did not find significant effects of the MOB program on the number of falls and recurrent fallers. Recurrent fallers may respond to interventions better than nonrecurrent fallers (20,21), which has been found in studies of the MOB program (2–4). However, only a few participants fell repeatedly during the short 8-week follow-up in the current study; therefore, the effects of the MOB program on frequent fallers may not have been evident. To further examine the effects of the MOB program on falls, long-term follow-up is recommended.

Older adults demonstrated significant improvement on the TUG test after completing the MOB program, extending the results of Ullmann et al (5). Furthermore, this study provided new evidence that the MOB program can improve older adults’ performance on the POMA and the FR test. However, 1 study indicated that the FR test may not discriminate between people who are at risk for falls and those who are not (22). Thus, future studies should examine the effects of the MOB program on other physical risk factors of falling and include trunk strength and flexibility in the FR test (23).

Booster training may be needed 6 months after the final session of the MOB program to maintain effects on psychological aspects of falling (1,2). Although our study found immediate effects of the MOB program on the POMA, the TUG test, and the FR test, the duration of these effects is unknown. Further investigation into whether a booster session is necessary to maintain these physical functions is needed.

There are limitations to this study. The study was not a randomized controlled trial and not blinded. The participants were self-selected, and Hawthorne Effects could have occurred. Participation could have led participants in each group to exercise harder or pay more attention to fall hazards. In addition, collecting falls data based on participants’ retrospective recall is potentially biased. We also recorded the number of physical activities participants described, but further details of the actual activities were not documented. Future studies should measure physical activities in terms of type, duration, and intensity (24). The results should be interpreted with caution given that the participants in the comparison group were younger and healthier compared with those in the MOB group. Although we statistically controlled for the differences at T1 between the 2 groups, a comparison group with the same characteristics as those in the MOB group would be optimal. However, most of these study limitations would likely result in an underestimation of the effects of the MOB program.

Unlike previous studies using single-group designs (3–5), this study included a comparison group, making the findings more robust. Overall, this study indicated that participation in the MOB program may reduce older adults’ immediate risk of falling and may improve their performance on the POMA, the TUG test, and the FR test. More studies are needed to examine the effects of the MOB program on recurrent falls and the number of falls. This study furthers our understanding of the MOB program on falls and physical risk of falling and supports the extended use of this program to improve physical risk of falling among older adults.
